# Oral arginine supplementation protects female mice from the onset of non-alcoholic steatohepatitis

**DOI:** 10.1007/s00726-017-2423-4

**Published:** 2017-04-22

**Authors:** Cathrin Sellmann, Christian Degen, Cheng Jun Jin, Anika Nier, Anna Janina Engstler, Dana Hasan Alkhatib, Jean-Pascal De Bandt, Ina Bergheim

**Affiliations:** 1 Institute of Nutritional Sciences, SD Model Systems of Molecular Nutrition, Friedrich-Schiller University Jena, Jena, Germany; 2 Department of Nutritional Sciences, Molecular Nutritional Science, University of Vienna, Althanstraße 14 (UZA II), 1090 Vienna, Austria; 3 Nutrition and Health Department, Faculty of Food and Agriculture, United Arab Emirates University, Abu Dhabi, United Arab Emirates; 4 EA4466, Sorbonne Paris Cité, and Clinical Chemistry Department, Hôpitaux Universitaires Paris Centre, APHP, Paris, France

**Keywords:** Arginine, Hepatic inflammation, Intestinal barrier function, Lipogenesis, Non-alcoholic steatohepatitis

## Abstract

Dietary arginine (Arg) supplementation has been proposed to have positive effects on the development of liver diseases. In the present study, we investigate if an oral Arg supplementation in diet protects mice fed a fructose, fat and cholesterol enriched Western-style diet (WSD) from the development of non-alcoholic steatohepatitis (NASH). Female C57BL/6J mice were fed a liquid control diet or a liquid WSD ± Arg (2.49 g/kg body weight/day) for 6 weeks. Indices of liver injury, glucose metabolism and intestinal permeability were determined. While Arg supplementation had no effects on body weight gain, fasting blood glucose levels were significantly lower in WSD+Arg-fed mice than in C+Arg-fed animals. WSD-fed mice developed liver steatosis accompanied with inflammation, both being significantly attenuated in WSD+Arg-fed mice. These effects of Arg supplementation went along with a protection against WSD-induced decreased tight junction protein levels in the upper parts of the small intestine, increased levels of bacterial endotoxin in portal plasma as well as increased hepatic toll-like receptor-4 mRNA and 4-hydroxynonenal protein adduct levels. In conclusion, Arg supplementation may protect mice from the development of NASH.

**Electronic supplementary material:**

The online version of this article (doi:10.1007/s00726-017-2423-4) contains supplementary material, which is available to authorized users.

## Introduction

Besides the metabolic syndrome, conditions like obesity, type 2 diabetes and dyslipidemia are also strongly associated with non-alcoholic fatty liver disease (NAFLD), which is increasingly becoming a worldwide health problem (for overview see Zhang and Lu 2015). Indeed, it has recently been suggested that in the United States NAFLD might even become the leading cause of liver transplantation by 2020 (Mahady and George 2016). Genetic predisposition, low physical activity and general overnutrition are accounted to be the main risk factors for the development of NAFLD (Liu 2012). However, results of human and animal studies suggest that dietary pattern and alterations in the intestine like changes in microbiota and barrier function, may also contribute to the development of the disease (Boursier et al. 2016; Tilg and Moschen 2010; Volynets et al. 2012). Mechanisms involved in the onset and progression of NAFLD are still not fully understood and universally accepted treatment and prevention strategies are not yet available (Mahady and George 2016; Rinella 2015).

The proteinogenic amino acid arginine (Arg) is found in a wide variety of foods such as dairy products and seafoods but also grains and legumes and is considered to be essential in human and animal nutrition (Hou and Wu 2017). Arg can be synthesized in vivo from glutamine, glutamate and proline via the intestinal-renal axis (Wu and Morris 1998) and it has been shown that Arg plays an integral role in the regulation of blood flow but also host defense (for overview see (Gogoi et al. 2015; Hou and Wu 2017). Indeed, nitric oxide (NO) produced during nitric oxide synthase (NOS)-mediated metabolism of Arg to citrulline plays a key role in inflammatory processes while ornithine being formed from Arg through arginase is a precursor for polyamines and proline, both being involved in tissue repair and cell proliferation (Rath et al. 2014). Oral Arg supplementation has been shown to attenuate lipopolysaccharide-induced inflammatory response (Tan et al. 2014) and to decrease bacterial translocation in the intestine in animal models (Quirino et al. 2013). However, whether an oral supplementation of Arg possesses protective effects against the onset of non-alcoholic steatohepatitis (NASH) has not yet been clarified. We hypothesized that via its role in inflammatory process and gut functions, Arg supplementation may limit the progression of early stages of NAFLD to NASH. Accordingly, the present study aimed to investigate if an oral Arg supplementation protects mice from the onset of a Western-style diet (WSD)-induced NASH and if so, to delineate responsible molecular mechanisms.

## Materials and methods

### Animals and treatment

As it has been shown before that female mice develop more pronounced liver damage, e.g., severe steatosis and signs of hepatic inflammation after 8–12 weeks of feeding a diet rich in fructose or different sugars and fat than male mice (Marin et al. 2016; Spruss et al. 2012a) and in the present study we aimed to determine the effects of Arg on the progression of steatosis to steatohepatitis, only female C57BL/6J mice (6–8 weeks old, Janvier S.A.S., Le-Genest-St-Isle, France) were used in the experiments. After all procedures were approved by the local Institutional Animal Care and Use Committee (IACUC), our experiments were carried out in a facility accredited by the Association for Assessment and Accreditation of Laboratory Animal Care (AAALAC). Mice (4 groups, *n* = 7–8) had free access to plain tap water at all times. For 6 weeks animals were pair-fed a liquid fructose-, fat-, and cholesterol-rich diet (Western-style diet, WSD; 17.8 MJ/kg dry diet: 60 E% carbohydrates, 25 E% fat, 15 E% protein with 50% wt/wt fructose and 0.16% wt/wt cholesterol, source of fat: butter, source of protein: casein) or a respective liquid control diet (*C*; 15.7 MJ/kg dry diet: 69 E% carbohydrates, 12 E% fat, 19 E% protein, source of fat: soybean oil, source of protein: casein) (Ssniff^®^, Germany) ± L-Arg (2.49 g/kg body weight (bw)/day) (Sigma-Aldrich Chemie GmbH, Germany, purity ≥98%) (C+Arg and WSD+Arg, respectively) as described previously and detailed in Online Resource Table 1 (Jin et al. 2015). In the mixed control diet, Arg concentration was 6.7 g/kg diet resulting in an average daily intake of 1.0 g Arg/kg bw, while in the control diet enriched with Arg the concentration was 23.8 g/kg diet resulting in an average daily intake of 3.5 g Arg/kg bw. In the mixed WSD diet, Arg concentration was 6.2 g/kg diet with an average daily intake of 0.8 g Arg/kg bw whereas in the mixed WSD enriched with Arg, concentration was 24.6 g/kg diet resulting in a daily intake of 3.3 g Arg/kg bw. To determine blood glucose levels, mice were fasted for 6 h and fasting blood samples were obtained from the retrobulbar venous plexus in the fifth week of feeding. Blood glucose levels were directly measured with a standard glucometer (Contour^®^, Bayer Vital GmbH, Germany). Anesthesia was performed with 100 mg of ketamine and 16 mg of xylazine/kg bw by intraperitoneal injection. Blood was collected from the portal vein just before killing and samples of liver as well as the upper part of the small intestine were snap-frozen or fixed in neutral-buffered formalin.

### Liver histology and clinical chemistry

As previously described in detail (Sellmann et al. 2015), sections of liver tissue embedded in paraffin (4 µm) were stained with hematoxylin and eosin to evaluate the NAFLD Activity Score (NAS). Staining for naphthol AS-D chloroacetate esterase (kit: Sigma–Aldrich Chemie GmbH, Steinheim, Germany) was used to determine the number of neutrophil granulocytes. Activities of alanine aminotransferase (ALT) and aspartate aminotransferase (AST) were measured in the routine laboratory at the University Hospital of Jena (Architect^®^, Abbott, Germany).

### Immunohistochemical staining of 4-HNE protein adducts in liver tissue and of the tight junction proteins occludin and ZO-1 in the upper part of the small intestine

4-Hydroxynonenal (4-HNE) protein adducts in paraffin embedded liver sections (4 µm) and tight junction proteins occludin and zonula occludens (ZO)-1 in intestinal tissue (4 µm sections) were stained using polyclonal antibodies (4-HNE: AG Scientific, USA; occludin: Invitrogen, USA; ZO-1: Invitrogen, USA). Extent of staining was determined as described previously (Sellmann et al. 2015). Briefly, data from eight randomly selected microscopic fields (200× for liver and 400× for intestine) of each tissue section were used to determine staining intensity.

### TNFα ELISA and endotoxin levels in portal plasma

Liver tissue was homogenized and protein concentrations of tumor necrosis factor (TNF) α were determined with a commercially available mouse TNFα kit following the instructions of manufacturer (Assaypro, St. Charles, USA). Endotoxin levels were measured in heparinized portal plasma as detailed before (Spruss et al. 2012b).

### RNA isolation and real-time RT-PCR

RNA isolation and real-time RT-PCR have been carried out as described previously (Spruss et al. 2012b). Primer sequences are summarized in Online Resource Table 2.

### Statistical analyses

All statistical analyses were performed using GraphPad Prism Software (La Jolla, USA). Results are shown as mean ± standard error of means (SEM). Before statistical analysis, outliers were identified using Grubb’s test and Bartlett’s test was performed to determine homogeneity of variances. Raw data were logarithmized in cases of unequal variances. Statistical significances between feeding groups were determined using two-way ANOVA with Tukey’s post hoc test (*P* ≤ 0.05) as no significant variances of homogeneity were found (*P* > 0.05).

## Results

### Effect of Arg supplementation on markers of apoptosis and injury in liver and body weight

**Fig. 1:**
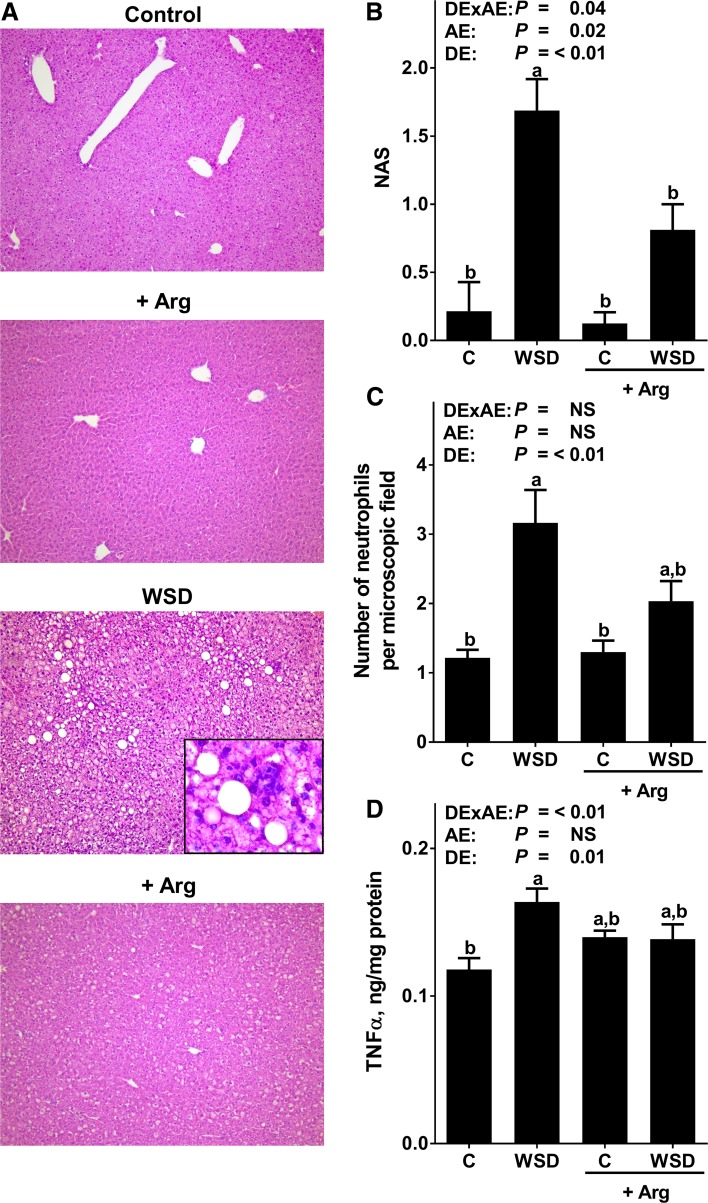
Indices of liver damage in female mice fed a C diet or WSD with or without Arg supplementation for 6 weeks. **a** Representative photomicrographs of hematoxylin and eosin staining of liver Sections (×100 and ×400). **b** Evaluation of liver histology using NAS. **c** Number of neutrophils in liver tissue. **d** Hepatic TNFα protein concentration. Values are mean ± SEM. Means without a common letter differ, *P* < 0.05. NS, *P* ≥ 0.05. Homogeneity of the variances was not significant (*P* > 0.05). *Arg* arginine; *C* control; *NAS* NAFLD activity score; *TNFα* tumor necrosis factor α; *WSD* Western-style diet; *DExAE* interaction between diet and arginine; *AE* arginine effect; *DE* diet effect

**Fig. 2:**
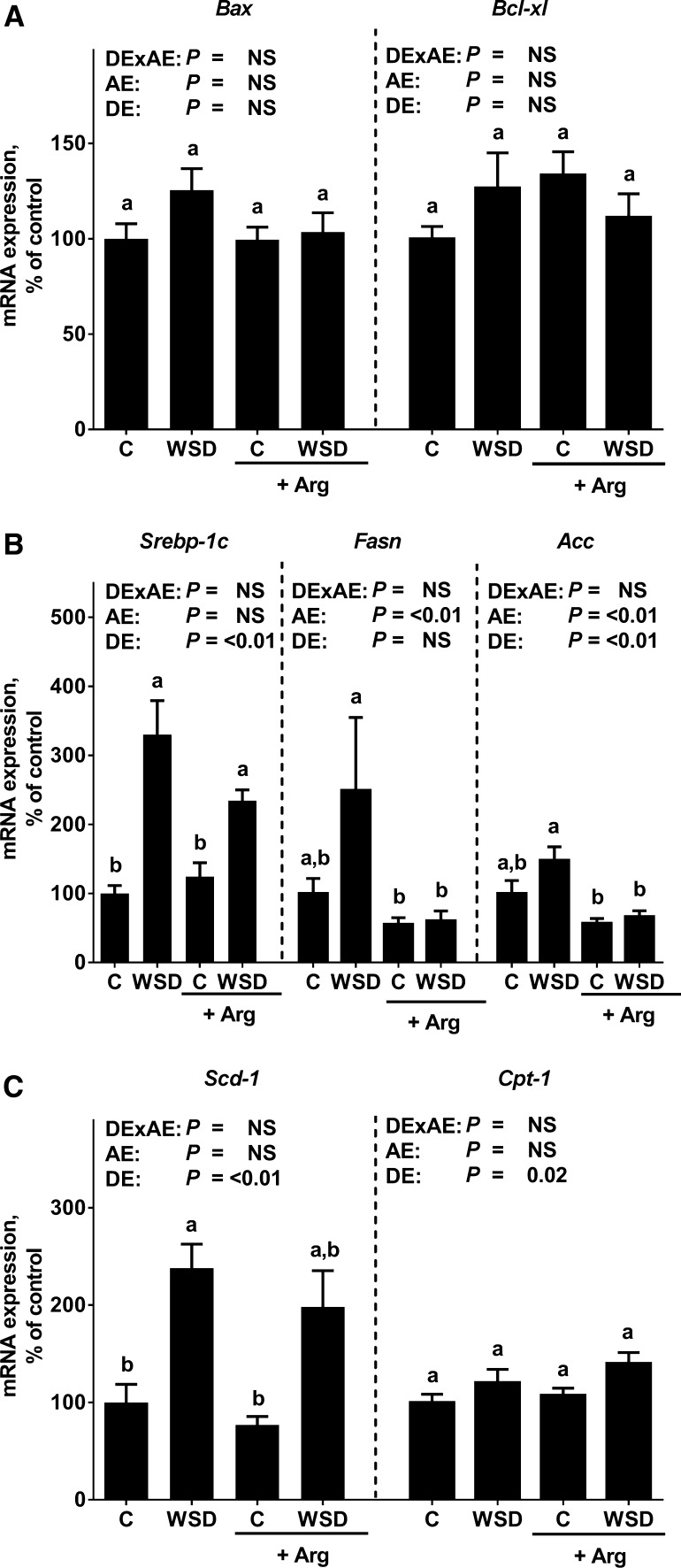
Hepatic markers of apoptosis and lipid metabolism in female mice fed a C diet or WSD with or without Arg supplementation for 6 weeks. Hepatic **a** *Bax* and *Bcl*-*xl*, **b** *Srebp*-*1c*, *Fasn* and *Acc* as well as **c** *Scd*-*1* and *Cpt*-*1* mRNA expression. Values are mean ± SEM. Means without a common letter differ, *P* < 0.05. NS, *P* ≥ 0.05. Homogeneity of the variances was not significant (*P* > 0.05). *Acc* acetyl-CoA carboxylase; *Arg* arginine; *Bax* BCL2-associated X protein; *Bcl*-*xl* B cell lymphoma extra-large; *C* control; *Cpt*-*1* carnitine palmitoyltransferase 1; *Fasn* fatty acid synthase; *Scd*-*1* stearoyl-CoA desaturase-1; *Srebp*-*1c* sterol regulatory element-binding protein 1c; *WSD* Western-style diet; *DExAE* interaction between diet and arginine, *AE* arginine effect, *DE* diet effect

Caloric intake, body weight gain and plasma ALT as well as AST activities were similar between all feeding groups. Absolute liver weight and liver to body weight ratios were significantly higher in both WSD-fed groups when compared to the two control groups (Table 1). Chronic intake of the WSD alone led to massive macrovesicular steatosis with beginning inflammation. In contrast, in mice fed the Arg-supplemented WSD hepatic steatosis but also signs of hepatic inflammation were significantly attenuated (NAS: WSD vs. WSD+Arg: *P* < 0.05). However, NAS for hepatic steatosis was still significantly higher in livers of WSD+Arg-fed mice when compared to the respective control group (scoring data not shown separately for steatosis) (Fig. 1). In line with these findings, number of neutrophils was also significantly higher in livers of WSD-fed mice when compared to controls. TNFα protein levels were significantly higher in livers of WSD-fed mice when compared to their respective control group, while protein levels of TNFα did not differ between groups fed diets supplemented with Arg. Neither mRNA expression of BCL2-associated X protein (*Bax*) nor B cell lymphoma extra-large (*Bcl*-*xl*) differed between groups (Fig. 2).

**Table 1:** Body and liver weights, plasma ALT and AST levels as well as markers of glucose homeostasis in blood and liver in female mice fed a C diet or WSD with or without Arg supplementation for 6 weeks

	Diet groups				*P* (2-factor ANOVA)^1^		
	*C*	WSD	C+Arg	WSD+Arg	DExAE	AE	DE
Caloric intake (kcal/mouse/day)	10 ± 0.1^a^	10 ± 0.2^a^	10 ± 0.1^a^	10 ± 0.1^a^	NS	NS	NS
Weight gain (g)	2.2 ± 0.2^a^	1.7 ± 0.2^a^	2.3 ± 0.3^a^	1.5 ± 0.4^a^	NS	NS	0.02
Absolute weight (g)	20.5 ± 0.4^a^	20.4 ± 0.2^a^	20.0 ± 0.5^a^	20.6 ± 0.6^a^	NS	NS	NS
Liver weight (g)	1.0 ± 0.1^b^	1.2 ± 0.0^a^	0.9 ± 0.0^b^	1.2 ± 0.0^a^	NS	NS	<0.01
Liver to body weight ratio (%)	5.0 ± 0.1^b^	6.0 ± 0.1^a^	4.7 ± 0.1^b^	5.7 ± 0.2^a^	NS	NS	<0.01
Plasma ALT (U/L)	16 ± 1.0^a^	23 ± 3.2^a^	16 ± 0.7^a^	20 ± 3.8^a^	NS	NS	NS
Plasma AST (U/L)	41 ± 1.9^a^	49 ± 4.2^a^	40 ± 1.7^a^	50 ± 4.5^a^	NS	NS	0.02
Blood glucose (mg/dL)	74 ± 5^b,c^	93 ± 5^a,b^	104 ± 5^a^	71 ± 5^c^	<0.01	NS	NS
Hepatic *Ir* mRNA (% of control)	100 ± 11^a^	105 ± 7^a^	103 ± 8^a^	111 ± 11^a^	NS	NS	NS
Hepatic *Irs*-*1* mRNA (% of control)	100 ± 17^a,b^	125 ± 24^a^	84 ± 16^a,b^	50 ± 6^b^	NS	<0.01	NS

Values represent mean ± SEM

Means without a common letter differ, *P* < 0.05. *NS P* ≥ 0.05

*ALT* alanine aminotransferase, *AST* aspartate aminotransferase, *Arg* arginine, *C* control, *Ir* insulin receptor, *Irs*-*1* insulin receptor substrate 1, *WSD* Western-style diet, *DExAE* interaction between diet and arginine, *AE* arginine effect, *DE* diet effect

^1^Homogeneity of the variances was not significant (*P* > 0.05)

### Effect of Arg supplementation on markers of glucose metabolism

In mice fed, the WSD, control diet and WSD+Arg, respectively, fasting blood glucose levels were similar. Fasting glucose levels of mice fed C+Arg were significantly higher than in mice fed plain control diet and WSD+Arg, respectively (+~40%, *P* < 0.05 in comparison to both groups). In liver tissue, expression of insulin receptor (*Ir*) was similar between groups whereas mRNA expression of insulin receptor substrate (*Irs*)-1 was significantly lower in livers of WSD+Arg-fed mice when compared to mice fed a plain WSD (Table 1).

### Effect of Arg supplementation on markers of hepatic lipid metabolism

Expression of sterol regulatory element-binding protein (*Srebp*)-1c mRNA in liver tissue was significantly higher in both WSD-fed groups regardless of additional treatment while expression did not differ between control groups (Fig. 2). In line with these findings, mRNA expression of stearoyl-CoA desaturase (*Scd*)-1 was also higher in livers of WSD-fed mice; however, as data varied considerably in some groups, level of significance was only reached in mice fed plain WSD when compared to the two control groups. In livers of WSD+Arg-fed mice *Scd*-*1* expression was only higher by trend when compared to the respective control group (*P* = 0.052). While mRNA expression of carnitine palmitoyltransferase (*Cpt*)-1 was similar between groups, mRNA expressions of acetyl-CoA carboxylase (*Acc*) and fatty acid synthase (*Fasn*) were significantly higher in livers of mice fed a plain WSD when compared to the two groups fed diets supplemented with Arg (+~60% for *Acc* and +~80% for *Fasn*, *P* < 0.05 for both groups) (Fig. 2).

### Effect of Arg supplementation on markers of intestinal barrier integrity and hepatic *Tlr*-*4* signaling

**Fig. 3:**
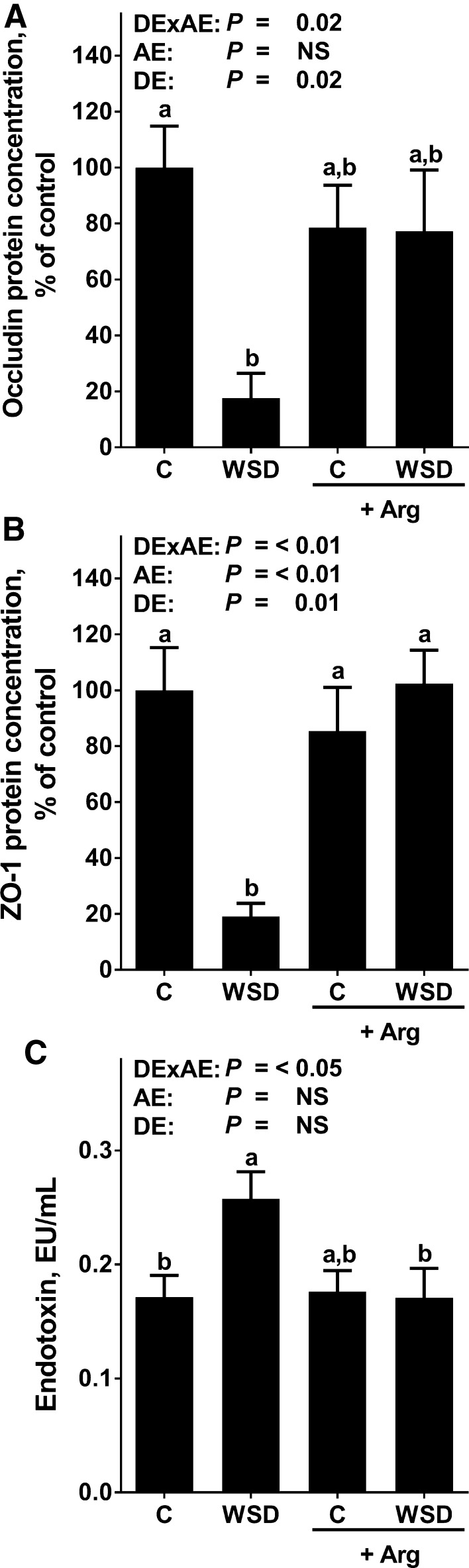
Markers of intestinal barrier function in female mice fed a C diet or WSD with or without Arg supplementation for 6 weeks. Densitometric analysis of **a** occludin and **b** ZO-1 protein staining in the upper parts of the small intestine, **c** endotoxin levels in portal plasma. Values are mean ± SEM. Means without a common letter differ, *P* < 0.05. NS, *P* ≥ 0.05. Homogeneity of the variances was not significant (*P* > 0.05). *Arg* arginine; *C* control; *WSD* Western-style diet; *ZO-1* zonula occludens 1; *DExAE* interaction between diet and arginine; *AE* arginine effect; *DE* diet effect

**Fig. 4:**
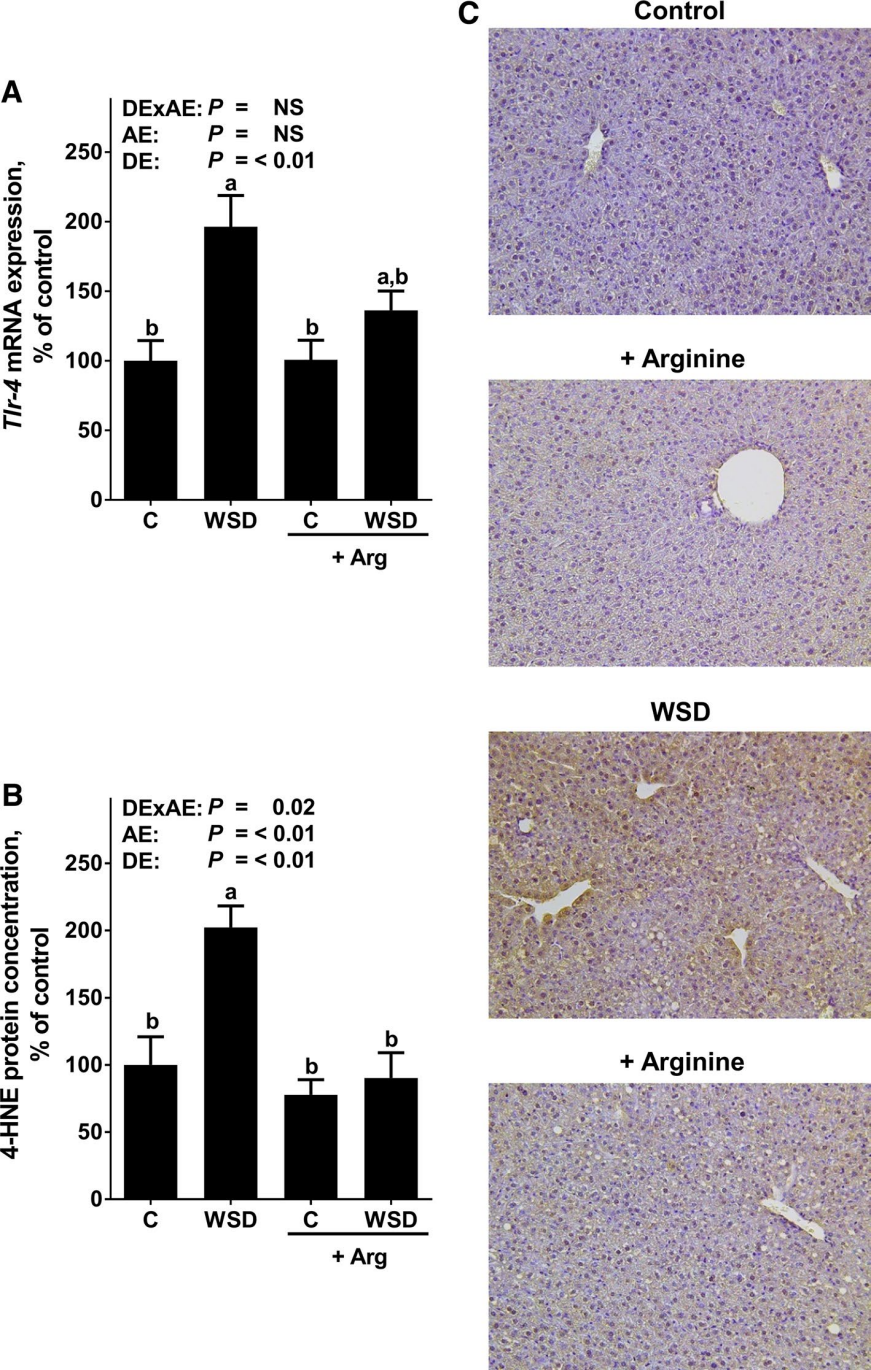
*Tlr*-*4* mRNA expression as well as 4-HNE protein adduct staining in female mice fed a C diet or WSD with or without Arg supplementation for 6 weeks. Hepatic **a** *Tlr*-*4* mRNA expression as well as **b** densitometric analysis and **c** representative photomicrographs of 4-HNE protein adduct staining in the liver. Values are mean ± SEM. Means without a common letter differ, *P* < 0.05. NS, *P* ≥ 0.05. Homogeneity of the variances was not significant (*P* > 0.05). *4-HNE* 4-hydroxynonenal; *Arg* arginine; *C* control; *Tlr*-*4* toll-like receptor 4; *WSD* Western-style diet; *DExAE* interaction between diet and arginine, *AE* arginine effect, *DE* diet effect

In line with findings of previous studies (Sellmann et al. 2016), protein levels of the tight junction proteins occludin and ZO-1 were significantly lower in the upper part of the small intestine of WSD-fed mice when compared to control diet-fed mice, whereas in WSD+Arg-fed mice they were at the level of controls (Fig. 3; Online Resource Figure 1). Portal endotoxin levels were also significantly higher in WSD-fed mice when compared to controls. In WSD+Arg-fed mice, this alteration was significantly attenuated. In line with these results, toll-like receptor (*Tlr*)-4 mRNA expression was significantly higher in livers of WSD-fed mice when compared to both control groups, whereas in livers of WSD+Arg-fed mice, mRNA expression of *Tlr*-*4* was almost at the level of controls (NS between control groups and WSD+Arg). Concentration of 4-HNE protein adducts was significantly higher in livers of plain WSD-fed groups when compared to all other groups. 4-HNE protein levels did not differ between control groups and WSD+Arg-fed mice (Fig. 4).

## Discussion

In the present study, in spite of similar total caloric intake and absolute body weight gain, we were able to show that an oral supplementation of Arg attenuates the development of WSD-induced NASH. Indeed, number of inflammatory foci, macrovesicular fat accumulation and number of neutrophils as well as expression of TNFα were all markedly lower in WSD+Arg-fed mice when compared to WSD-fed animals. However, as mice only developed early signs of NASH, ALT and AST levels were not elevated. Others have shown before that treating rodents with Arg in genetic or diet-induced models of obesity is associated with decreased weight gain and white adipose tissue mass, whereas brown adipose tissue and skeletal muscle mass were increased (Fu et al. 2005; Jobgen et al. 2009a, b; Wu et al. 2012) and for overview Wu and Morris 1998). In the present study, using an isocaloric feeding model, similar effects on bw were not found. However, as fat mass was not determined in the present study, it cannot be ruled out that the beneficial effects of the Arg supplementation at least in part might have resulted from changes in body composition, e.g., an increase in brown adipose tissue fat mass. Effects of supplementing Arg on adipose tissue and skeletal muscle in isocaloric feeding models will have to be determined in future studies. Furthermore, no modifications in insulin sensitivity were observed. Indeed, in the present study, fasting blood glucose levels and hepatic mRNA expression of *Ir* and *Irs*-*1* were similar between WSD and control group; however, expressions as well as blood glucose levels varied considerable within groups. Interestingly, blood glucose levels were higher in the C+Arg group compared to the controls and the WSD+Arg-fed mice. This is in contrast to the findings of Jobgen et al. in high fat diet-fed rats showing that supplementing 1.51% Arg-HCl in drinking water lowered fasting glucose levels (Jobgen et al. 2009a). Differences between the present study and that of Jobgen et al. might have resulted from differences in the diets fed, e.g., high fat diet vs. WSD and species used (mouse vs. rat) as well as experimental design. Effects of Arg supplementation on glucose metabolism in different settings as well as mechanisms involved will have to be determined in further studies. Our data are also not in line with previous findings of Jegatheesan et al. showing that an oral Arg supplementation was not associated with a protection of rats against fructose-induced steatosis (Jegatheesan et al. 2016). However, differences between the present study and that of Jegatheesan et al. might have resulted from differences in the animal models used, e.g., rats fed a high fructose diet for 4 weeks vs. mice fed a fructose, fat and cholesterol-rich diet for 6 weeks in the present study and the investigated disease stages (in the present study: early phases of NASH vs. onset of steatosis in the study of Jegatheesan et al. 2016). In summary, our data suggest that an oral Arg supplementation attenuated the development of early stages of NASH in mice induced by feeding a fructose, fat and cholesterol-rich diet. However, as in the present study, no isonitrogenous group was included and plasma Arg as well as total amino acid profile was not determined, it cannot be excluded that differences found might have resulted from the higher protein intake found in Arg-treated mice (WSD: 15 E% protein vs. WSD+Arg: 16.5 E% protein) and a resulting altered amino acids plasma profile. This will have to be addressed in future studies.

### The protective effects of an oral Arg supplementation are associated with some changes of markers of hepatic lipid metabolism

Results of several studies suggest that an altered hepatic lipid metabolism, e.g., an increased de novo lipid synthesis and insufficient fatty acid oxidation as well as triglyceride secretion is critical in the development of NAFLD (for overview see Kawano and Cohen 2013). Indeed, it has been shown that the inhibition of de novo lipogenesis and *Srebp*-1c-dependent signaling cascades and the activation of fatty acid β-oxidation may exert beneficial effects on the development of NAFLD in vitro and in rodent models (Rodriguez-Ramiro et al. 2016). Supplementation of Arg but even more so of its endogenous metabolite agmatine, has been suggested to modulate markers of lipogenesis, e.g., to suppress expression of *Srebp*-*1c* and *Fasn* in different tissues (Sharawy et al. 2016; Tan et al. 2011). Also, probably through altering bioavailability of NO, Arg has been shown to alter expression of genes involved in energy metabolism and particularly β-oxidation (Jobgen et al. 2009a, 2006). In the present study, *Srebp*-*1c* mRNA expression was markedly higher in livers of both WSD-fed groups while expressions of *Fasn*, *Acc* and *Scd*-*1*, all known to be strongly regulated through *Srebp*-*1c*-dependent mechanisms (Serviddio et al. 2013), were markedly higher in livers of mice only fed a WSD. Indeed, all three markers seemed to be affected by the supplementation of Arg regardless of diet fed and independently of *Srebp*-*1c,* as the latter seemed not to be differently regulated at the level of mRNA expression when compared to mice not fed Arg. However, as *Srebp*-*1c* activity is not only regulated at the level of transcription (Lai et al. 2016), it cannot be ruled out that *Srebp*-*1c* activity might have been affected by the supplementation of Arg. Our results are in part contrasting the results of Jobgen et al. (2009a) who did not report alterations in controls for *Acc, Fasn* or *Scd*-*1* in adipose tissue of rats treated with Arg while animals being fed a high fat diet were affected by the Arg supplementation. However, differences between our results and those of Jobgen et al. might have resulted from differences in diets used and study design as well as species studied and tissues analyzed, e.g., adipose tissue vs. liver tissue. Future studies will have to determine how Arg affects *Srebp*-*1c* and dependent molecules. In contrast, expression of *Cpt*-*1* was not different between groups, suggesting that contrary to the findings of others in settings of high fat diet-induced NAFLD (Lai et al. 2016), the development of NAFLD in the present study was not associated with marked alterations of long chain fatty acid metabolism. Again, differences between studies of others and our own might have resulted from differences in experimental setup but also detection methods used (e.g., real-time PCR vs. Western blot) (Lai et al. 2016; Sharawy et al. 2016; Tan et al. 2011). Taken together, our data suggest that the beneficial effects of an Arg supplementation on the development of NASH found in the present study may at least in part have resulted from alterations of hepatic lipogenesis. However, molecular mechanisms involved in the effects of Arg on hepatic *Fasn*, *Acc* and *Scd*-*1* expression remain to be determined.

### Oral Arg supplementation attenuates the increased translocation of intestinal bacterial endotoxin found in WSD-fed mice

Alterations of intestinal microbiota composition and barrier function resulting in an increased permeation of bacterial endotoxin into the portal blood have repeatedly been shown to be associated with the development of NAFLD (for overview see Abdul-Hai et al. 2015; Kirpich et al. 2015). Furthermore, studies suggest that an improvement of liver status in patients with NAFLD is associated with a decrease of peripheral blood endotoxin levels (Volynets et al. 2012). Indeed, it has been shown that targeting intestinal barrier integrity attenuates the development of liver damage in mouse and rat models of NAFLD and NASH (Ritze et al. 2014; Spruss et al. 2012b). Arg is a critical factor in the regulation of intestinal barrier function (Costa et al. 2014; Gogoi et al. 2015); however, molecular mechanisms underlying the protective effects of Arg on intestinal barrier function have not yet been fully understood. Indeed, it has been discussed that Arg may alter intestinal microbiota composition and metabolism but also NO synthesis (for overview also see Blachier et al. 2011; Gogoi et al. 2015). Here, the beneficial effects of Arg on the development of beginning NASH were associated with a protection against the WSD-induced loss of tight junction proteins in the upper parts of the small intestine and with an increased translocation of bacterial endotoxins into portal plasma. In line with previous findings of our group (Spruss et al. 2012b), the “normalization” of portal endotoxin levels found in mice fed WSD+Arg was associated with a protection against the induction of *Tlr*-*4* mRNA expression and 4-HNE protein adducts levels in the liver. Our findings are also in line with other studies using cell culture and animals models indicating that a treatment with Arg may prevent the loss of the tight junction proteins occludin and ZO-1 and subsequently improve intestinal barrier function (Beutheu et al. 2013; Ren et al. 2014). Results of these studies also suggest that the beneficial effects of Arg supplementation on intestinal barrier function involve mechanisms dependent on NO donation and immune function (Chapman et al. 2012; Wang et al. 2015). If similar mechanisms were involved in the beneficial effects of Arg supplementation in the present study remains to be determined. Taken together, results of the present study suggest that an oral Arg supplementation may have protective effects on early stages of NASH development and that this is associated with a protection against the loss of tight junction proteins in the small intestine, the increased permeation of endotoxin into the portal blood and induction of *Tlr*-*4*-dependent signaling cascades in the liver. However, molecular mechanisms underlying the effects of Arg on intestinal barrier integrity remain to be determined.

## Conclusion

In summary, our data suggest that an oral Arg supplementation at least partially protects mice from the development of WSD-induced early signs of NASH. Results of our study also suggest that the beneficial effects of Arg may have resulted from a protection against the enhanced intestinal permeability and subsequently increased permeation of bacterial endotoxins into the portal blood found in this model of NASH. However, several findings of others suggest that a supplementation of Arg may also affect many other metabolic and immunological functions (for overview see Jobgen et al. 2006; Wu and Morris 1998). Therefore, further studies are needed to delineate molecular mechanisms responsible for the beneficial effects of Arg on intestinal barrier function and metabolism during NAFLD progression and to determine if similar effects are also found in humans.

## Electronic supplementary material

Below is the link to the electronic supplementary material.

Supplementary material 1 (PDF 89 kb)

Supplementary material 2 (PDF 71 kb)

Supplementary material 3 (PDF 361 kb)

## Abbreviations

4-HNE: 4-Hydroxynonenal
*Acc*: Acetyl-CoA carboxylase
ALT: Alanine aminotransferase
Arg: Arginine
AST: Aspartate aminotransferase
*Bax*: BCL2-associated X protein
*Bcl*-*xl*: B cell lymphoma extra-large
*Cpt*-*1*: Carnitine palmitoyltransferase 1
*Fasn*: Fatty acid synthase
NO: Nitric oxide
NOS: Nitric oxide synthase
*Ir*: Insulin receptor
*Irs*-*1*: Insulin receptor substrate 1
NAFLD: Non-alcoholic fatty liver disease
NAS: NAFLD Activity Score
NASH: Non-alcoholic steatohepatitis
*Scd*-*1*: Stearoyl-CoA desaturase-1
SPF: Specific pathogen free
*Srebp*-*1c*: Sterol regulatory element-binding protein 1c
*Tlr*-*4*: Toll-like receptor 4
TNFα: Tumor necrosis factor α
WSD: Western-style diet
ZO-1: Zonula occludens-1
