# Supplementary material for: Oral arginine supplementation protects female mice from the onset of non-alcoholic steatohepatitis
Source: Amino Acids. 2017 Apr 22;49(7):1215–25. doi: 10.1007/s00726-017-2423-4 (PMC5487836; doi:10.1007/s00726-017-2423-4)
Supplement: Supplementary file 1 — Supplementary material 1 (PDF 89 kb) [file 726_2017_2423_MOESM1_ESM.pdf]

Oral arginine supplementation protects female mice from the onset of non-alcoholic steatohepatitis, Amino Acids, Sellmann C, Degen C, Jin CJ, Nier A, Engstler AJ, Hasan Alkhatib D, De Bandt JP, Bergheim I. E-Mail: ina.bergheim@univie.ac.at, University of Vienna

Online Resource Table 1. Nutrient composition of control or Western-style diet fed female mice for 6 weeks (dry diet).

		Control diet	Western-style diet
Crude protein, CP	% wt/wt	17.4	16.0
Crude fat, CL	% wt/wt	5.1	11.8
Crude fibre, CF	% wt/wt	5.0	2.0
Crude ash	% wt/wt	4.1	4.2
Starch	% wt/wt	34.6	5.0
Sucrose	% wt/wt	11.0	-
Glucose	% wt/wt	-	5.0
Fructose	% wt/wt	-	50.0
Cholesterol	% wt/wt	-	0.16
L-Lysine	% wt/wt	1.43	1.32
L-Methionine + L-Cysteine	% wt/wt	1.00	0.96
L-Threonine	% wt/wt	0.75	0.69
Calcium	% wt/wt	0.78	0.77
Phosphorus	% wt/wt	0.48	0.47
Sodium	% wt/wt	0.23	0.30
Vitamin A (retinol acetate)	IU/kg	15000	15000
Vitamin D_3 (cholecalciferol)	IU/kg	1500	1500
Vitamin E (α-tocopherol acetate)	mg/kg	150	150
Fatty acids			
4:0	% wt/wt	-	0.44
6:0	% wt/wt	-	0.29
8:0	% wt/wt	-	0.16
10:0	% wt/wt	-	0.35
12:0	% wt/wt	-	0.40
14:0	% wt/wt	0.02	1.23
16:0	% wt/wt	0.57	3.10
18:0	% wt/wt	0.18	1.14
18:1 (n-9)	% wt/wt	1.28	2.58
18:2 (n-6)	% wt/wt	2.65	0.21
18:3 (n-3)	% wt/wt	0.29	0.06
Metabolizable energy (ME)	MJ/kg	15.7	17.8
CP	kJ%	19	15
CL	kJ%	12	25
Carbohydrate, CHO	kJ%	69	60
