# Supplementary material for: Oral arginine supplementation protects female mice from the onset of non-alcoholic steatohepatitis
Source: Amino Acids. 2017 Apr 22;49(7):1215–25. doi: 10.1007/s00726-017-2423-4 (PMC5487836; doi:10.1007/s00726-017-2423-4)
Supplement: Supplementary file 2 — Supplementary material 2 (PDF 71 kb) [file 726_2017_2423_MOESM2_ESM.pdf]

Oral arginine supplementation protects female mice from the onset of non-alcoholic steatohepatitis, Amino Acids, Sellmann C, Degen C, Jin CJ, Nier A, Engstler AJ, Hasan Alkhatib D, De Bandt JP, Bergheim I. E-Mail: ina.bergheim@univie.ac.at, University of Vienna

Online Resource Table 2. Primer sequences used for real-time RT-PCR to determine expression of genes in liver tissue in female mice fed a C diet or WSD with or without Arg supplementation for 6 weeks.[1]

Gene name	Forward (5' – 3')	Reverse (5' – 3')
18S	gta acc cgt tga acc cca tt	cca tcc aat cgg tag tag cg
Acc	ctt cct cct gat gag caa ctct	cgt gag ttt tcc caa aat aag c
Bax	tgc aga gga tga ttg ctg ac	gat cag ctc ggg cac ttt ag
Bcl-xl	atg gca gca gtg aag caa g	gca atc cga ctc acc aat acc
Cpt-1	gtg ttg gag gtg aca gac tt	cac ttt ctc ttt cca caa gg
Fasn	tct ggg cca acc tca ttg gt	gaa gct ggg ggt cca ttg tg
Ir	cat ccc gaa agc gaa gat cc	gag tcc tga ttg cat gcc tgc ag
Irs-1	gct cta gtg ctt ccg tgt cc	gtt gcc acc cct aga caa aa
Scd-1	ccg ata aaa ggg ggc tga gg	tgc tga gat cga gcg tgg ac
Srebp-1c	acc ggc tac tgc tgg act gc	aga gca aga ggg tgc cat cg
Tlr-4	agc cat tgc tgc caa cat ca	gct gcc tca gca gga ctt c

[1]*18S* = 18S ribosomal RNA, *Acc* = acetyl-CoA carboxylase, *Bax*, BCL2-associated X protein; *Bcl-xl*, B-cell lymphoma extra-large; *Cpt-1* = carnitine palmitoyltransferase 1, *Fasn* = fatty acid synthase, *Ir* = insulin receptor, *Irs-1* = insulin receptor substrate 1, *Scd-1* = stearoyl-CoA desaturase, *Srebp-1c* = sterol regulatory element-binding protein 1c, *Tlr-4* = toll-like receptor 4.
