# Supplementary material for: Oral arginine supplementation protects female mice from the onset of non-alcoholic steatohepatitis
Source: Amino Acids. 2017 Apr 22;49(7):1215–25. doi: 10.1007/s00726-017-2423-4 (PMC5487836; doi:10.1007/s00726-017-2423-4)
Supplement: Supplementary file 3 — Supplementary material 3 (PDF 361 kb) [file 726_2017_2423_MOESM3_ESM.pdf]

Oral arginine supplementation protects female mice from the onset of non-alcoholic steatohepatitis, Amino Acids, Sellmann C, Degen C, Jin CJ, Nier A, Engstler AJ, Hasan Alkhatib D, De Bandt JP, Bergheim I. E-Mail: ina.bergheim@univie.ac.at, University of Vienna

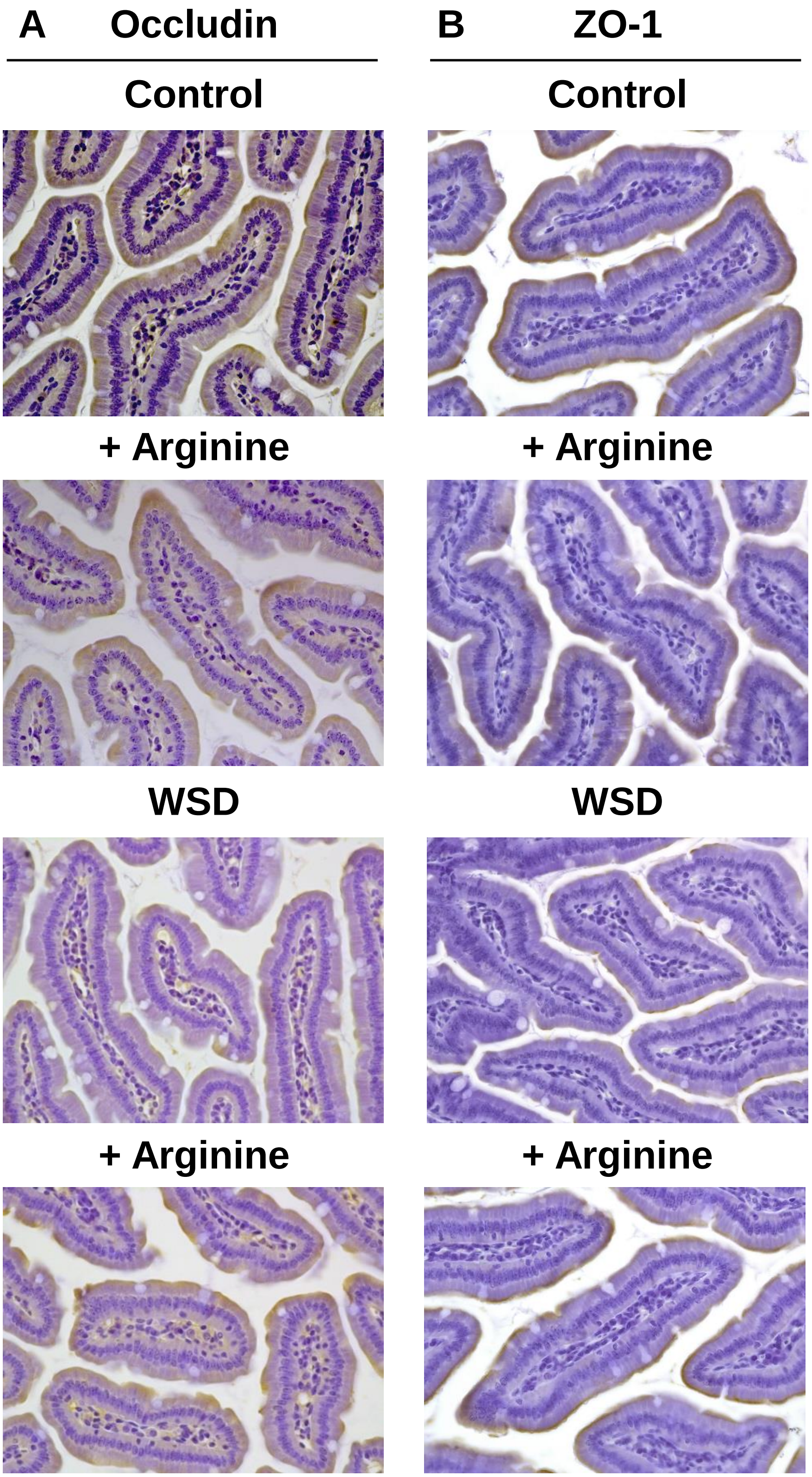

Supplemental Figure 1. Staining for tight junction proteins in the small intestine of female mice fed a C diet or WSD with or without Arg supplementation for 6 weeks. Representative photomicrographs of (A) occludin and (B) ZO-1 staining in upper parts of the small intestine (400x).

Supplemental Figure 1 – Sellmann *et al*.
